# Supplementary material for: Thermally Conductive and Electrical Insulation BNNS/CNF Aerogel Nano-Paper
Source: Polymers (Basel). 2019 Apr 10;11(4):660. doi: 10.3390/polym11040660 (PMC6523969; doi:10.3390/polym11040660)
Supplement: Supplementary file 1 [file polymers-11-00660-s001.pdf]

Supporting Information

Thermally Conductive and Electrical Insulation BNNS/CNF Aerogel Nano-paper

Xiu Wang[1,2], Zhihuai Yu[1], Huiyang Bian[1], Weibing Wu[1], Huining Xiao,[1,2,*] Hongqi Dai[1,*]

[1] Jiangsu Co-Innovation Center of Efficient Processing and Utilization of Forest Resources, Nanjing Forestry University, Nanjing , 210037, China

[2] Department of Chemical Engineering, University of New Brunswick, Fredericton, NB, E3B 5A3. Canada

Correspondence: hgdhq@njfu.edu.cn(H. D.)； hxiao@unb.ca(H. X.)

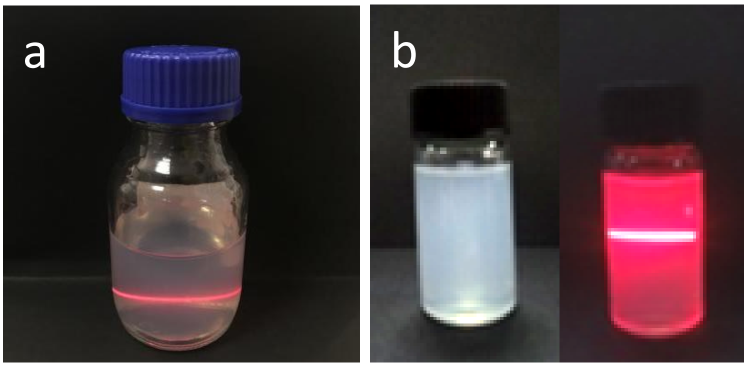

Figure S1. (a) Tyndall phenomenon of CNF. (b) Optical photograph (left) and Tyndall phenomenon (right) of BNNS.

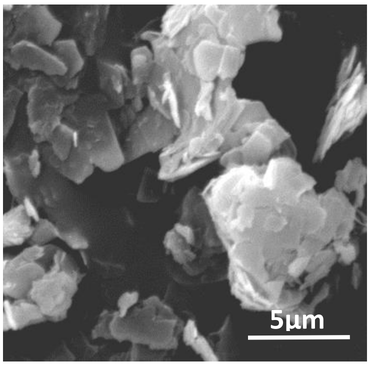

Figure S2. SEM image of commercial h-BN.

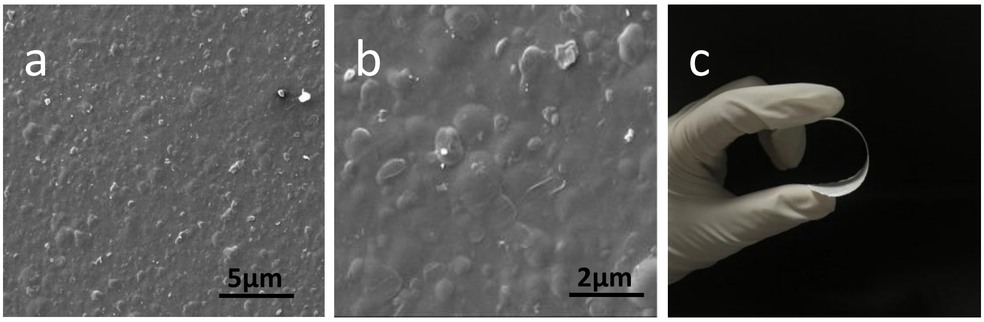

Figure S3. (a) and (b) SEM images under different meganifications of BNNS/CNF aerogel nano-paper. (c) Optical photograph of BNNS/CNF aerogel nano-paper about good bending and folding properties.

Table S1. Comparing the specific heat, density and thermal diffusivity of BNNS /CNF nano-paper prepared by different methods.

BNNS Content (wt%)	Temperature (℃)	Blended nano-paper			Aerogel template nano-paper		
		Specific Heat J/gK	Density g/cm^3	Thermal Diffusivity mm^2/s	Specific Heat J/gK	Density g/cm^3	Thermal Diffusivity mm^2/s
0	20	1.489 ±0.005	1.035 ±0.005	0.268 ±0.006	1.489 ±0.005	1.035 ±0.006	0.268 ±0.004
10	20	1.414 ±0.005	1.115 ±0.007	0.277 ±0.003	1.409 ±0.005	1.120 ±0.004	0.556 ±0.005
20	20	1.354 ±0.007	1.196 ±0.007	0.339 ±0.003	1.357 ±0.004	1.194 ±0.005	0.694 ±0.005
30	20	1.248 ±0.004	1.279 ±0.007	0.425 ±0.005	1.239 ±0.005	1.287 ±0.008	0.827 ±0.006
40	20	1.237 ±0.004	1.358 ±0.003	0.472 ±0.006	1.219 ±0.008	1.378 ±0.008	0.912 ±0.006
50	20	1.121 ±0.005	1.440 ±0.005	0.537 ±0.003	1.132 ±0.006	1.426 ±0.008	1.040 ±0.006
